# An Investigation into Soft Error Detection Efficiency at Operating System Level

**DOI:** 10.1155/2014/506105

**Published:** 2014-01-19

**Authors:** Seyyed Amir Asghari, Okyay Kaynak, Hassan Taheri

**Affiliations:** ^1^Amirkabir University of Technology, Tehran 158754413, Iran; ^2^Harbin Institute of Technology, Harbin 15000, China; ^3^Bogazici University, Istanbul 80815, Turkey

## Abstract

Electronic equipment operating in harsh environments such as space is subjected to a range of threats. The most important of these is radiation that gives rise to permanent and transient errors on microelectronic components. The occurrence rate of transient errors is significantly more than permanent errors. The transient errors, or soft errors, emerge in two formats: control flow errors (CFEs) and data errors. Valuable research results have already appeared in literature at hardware and software levels for their alleviation. However, there is the basic assumption behind these works that the operating system is reliable and the focus is on other system levels. In this paper, we investigate the effects of soft errors on the operating system components and compare their vulnerability with that of application level components. Results show that soft errors in operating system components affect both operating system and application level components. Therefore, by providing endurance to operating system level components against soft errors, both operating system and application level components gain tolerance.

## 1. Introduction

Embedded system designers have two options for selecting electronic equipment that will operate in harsh environments such as space [1–12]: (i) High Reliability (Radiation Tolerant or Radiation Hardened) equipment and (ii) Commercial Off-The-Shelf (COTS) equipment. High Reliability equipment has high reliability value but is costly and its performance in comparison with COTS equipment is low. The more common approach in space missions is the use of COTS equipment (such as COTS evaluation boards). However, COTS equipment carries considerable reliability concerns, as they are vulnerable against radiation that is the most important threat in space environment. Therefore, the reliability of COTS equipment should be increased with hardware and software reliability enhancement methods [11, 12].

The most important type of faults caused by radiation in space is Single Event Upsets (SEU) that interferes with the behavior of microelectronic devices. The SEU changes the state of microelectronic devices such as semiconductor memories and processor registers, due to heavy ions and electromagnetic radiations. With the recent reductions in the size of transistors in manufacturing process, the impact of radiation on microelectronic modules has become more prominent [11–28], that caused a higher rate of SEU occurrence.

Faults that occur in electronic systems can be categorized into two as transient and permanent faults. The occurrence rate of transient faults is significantly more than the other. Transient faults in turn are converted into two error types: those that can illegally change the flow of the program running on the system and those that change the data content. The first type is called control flow error (CFE) and the other is called data error. The experimental research indicates that about 33% to 77% of transient faults are converted to CFEs and the remaining ones are converted into data errors. The CFEs and the data errors are collectively called soft errors or transient errors [11–18].

Various valuable approaches have been proposed in literature for the management (detection and correction) of soft errors. However they all are for application and hardware levels. The use of hardware methods needs hardware reconfigurations and therefore, in most applications that utilize COTS equipment, these methods are not the most appropriate options [11–15].

In multitask embedded systems, the utilization of an operating system is essential in many cases. It can manage important system parameters such as memory and time. In the application of soft error management methods at system level, the operating system has until now been neglected. It must be noted that the effects of soft errors on operating system components can have dramatic impacts not only at operating system level but also at application level.

The authors have previously published some papers on soft error management, all of which, like all the other software methods, have been applied in application level. In this paper, three significant methods previously developed by the authors, namely, Software-based Control Flow Checking (SCFC) [11], Control Checking Method for Multitask Environment (CFCME) [12], and Critical Path Duplication (CPD) [16] are used on operating system level and their performance is evaluated.

The motivation behind this paper is that no research can be seen in literature that proposes measures against soft errors at operating system level. The study focuses on

- investigation of CFEs effects on operating system level components,
- investigation of data error effects on operating system level components,
- comparison of vulnerability of operating system and application level components to soft errors.

## 2. Previous Works

Electronic equipment that operates in industrial environments, especially in harsh environments such as in space missions, is subjected to transient faults due to radiations (gamma-rays, X-rays, proton, neutron, and energetic photons). These transient faults that are also called soft errors are in turn converted to CFEs and data errors. For the management of soft errors, many methods have been proposed since the 1980s [11–28].

**Figure 1:**
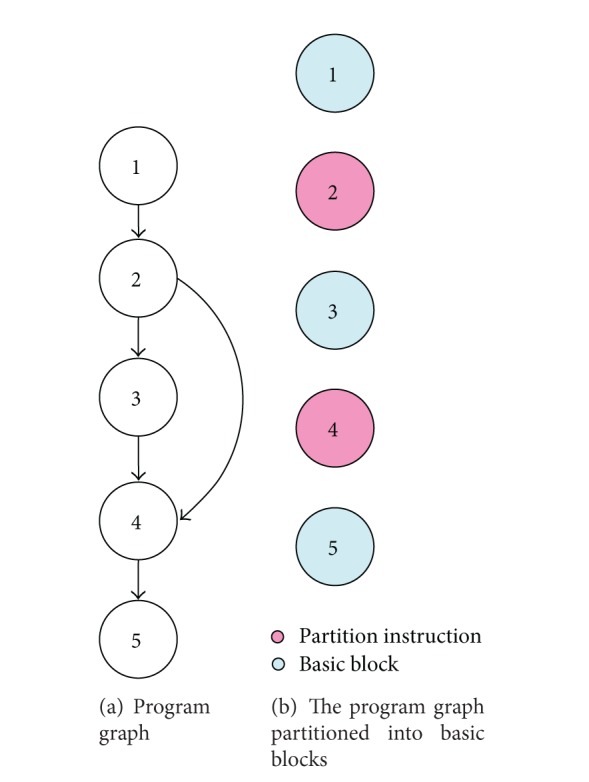
Program dividing into some basic blocks [11].

Control Flow Checking (CFC) methods can be categorized into two as hardware and software methods. In all, the original program is first divided into basic blocks (BB) which form the Control Flow Graph (CFG). The BB is a segment of the program such that its internal instructions are run serially without any jumps. Therefore, a jump or a branch instruction cannot be a part of a BB. A simple program and its related CFG are shown in Figure 1 as an example [11].

In CFC methods, the BBs have unique signature value that changes in program flow. The designer can assign the signatures to BBs or they can be derived from other BBs.

**Figure 2:**
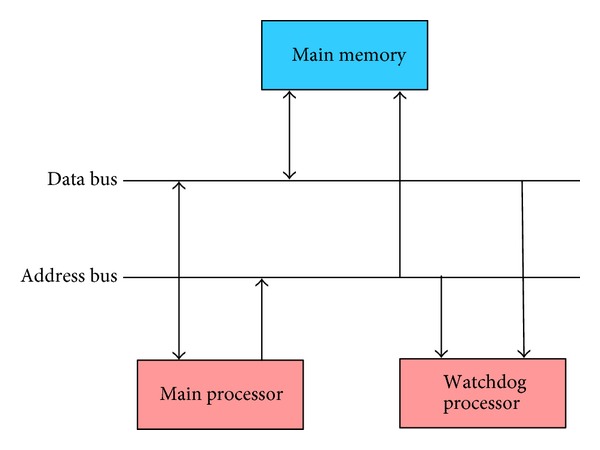
Hardware Control Flow Checking with a Watchdog processor [11–13].

In hardware CFC methods, a Watchdog processor (WP) is utilized for monitoring the correct changes of signature in transition from a BB to the successor BB. The successor BB is the next BB in CFG. The previous BB in CFG is called the predecessor BB. The main structure of hardware CFC that utilizes a WP is shown in Figure 2. The WP in this structure has the CFG, as it should be. It also has the correct signatures derived by a run (the golden run). Since the WP and the main processor share the same buses, the WP can, during a real run, monitor the flow changes of the program. Any mismatch between the real run signatures and the golden run signatures generates an error signal [11–13].

Software CFC methods differ from the hardware ones by the significant difference that, in the software CFC methods, CFC is done by instructions that are embedded in the original program.

## 3. The Highlights of SCFC, CFCME, and CPD Methods

In this section, a brief review of the previously mentioned SCFC, CFCME, and CFP methods from the literature is given. All of these methods can be applied to the components of the operation system kernel.

### 3.1. A Brief Review of the SCFC Method

The SCFC (Software-based Control Flow Checking) [11] method is a CFE detection method for use in single task processing systems. It has the detection capability of both inter- and intrabasic block control flow errors. The SCFC delivers a better Evaluation Factor (EF) [11] parameter in comparison of other well-known software-based methods (CFCSS [17], ECCA [22], and RSCFC [23]). In order to detect CFEs, four instructions are added to the original program instructions: *Control*, *Check*, *Update*, and *Exit*.

Each BB in this method has a unique ID number (the unique number of each BB in CFG) and a unique signature value (this value has n bits where n is the number of BB in CFG). The signature of each BB is updated in the middle of BB after the *Update* instruction. The ID number is updated at the end of the BB, after the *Exit* instruction. In updating the ID number, this number is equaled to the ID number of the successor BB or BBs. In updating the signature, the bit of the corresponding successor or successors of current BB is set to 1.

The *Control *instruction that is inserted at the top of each BB compares the ID of the current BB with the ID or IDs arriving from the predecessor BB. The current basic block ID should be seen in the ID list that reaches from the predecessor BB. If not, a signal is generated. This signal shows a CFE.

The *Check* instruction checks the value of the IDth bit of the current BB. It should be one. If not, an error signal is generated. This signal indicates a CFE.

The *Update* instruction updates the current BB signature. In this updating procedure, as mentioned before, the IDth bit of the successor BB or BBs is set to 1.

The *Exit* instruction updates the ID value by equaling it to the ID number of the successor BB or BBs.

**Figure 3:**
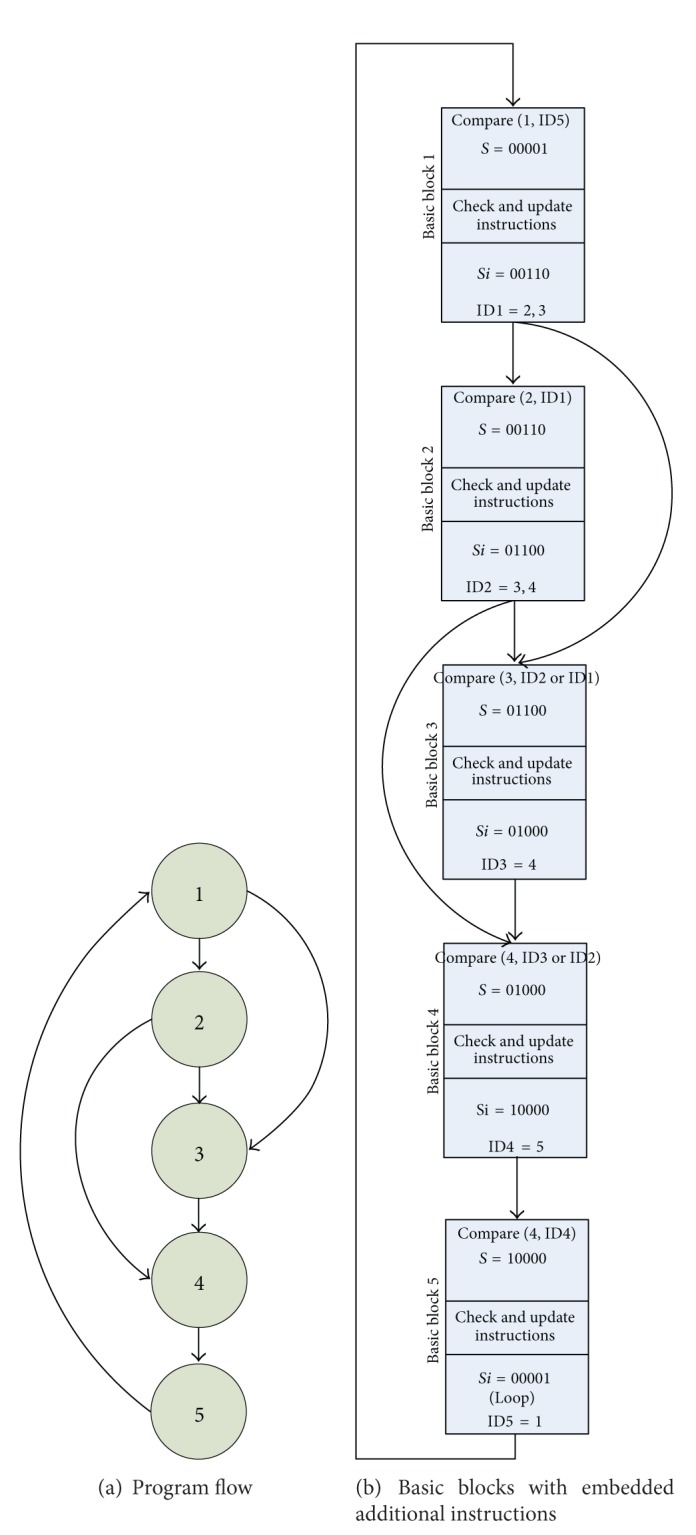
An example of the SCFC operations for control flow error detection.

Figure 3 shows an example of the SCFC operations for control flow error detection.

### 3.2. A Brief Review of the CFCME Method

The CFCME (Control Checking Method for Multitask Environment) [12] method is the improved version of the SCFC for multitask processing environment. The overall structure of the operation and the instructions of the CFCME method is the same as those in SCFC method. The main difference between these two methods is that in the CFCME method a code is utilized instead of a signature. The left part of the code bit stream is assigned to the thread or the task number and the right part is assigned to the unique signature value of each basic block. The number of bits in the left part of the code equals to ⌊log_2_^*T*^⌋ + 1, where *T* is the task or the thread number of the system. The number of bits of the right part equals to that of the basic block number of the CFG. The assignment and the updating procedure of the CFCME method are similar to the SCFC method. In the CFCME method, three instructions are added to each BB: (i) Check, (ii) Update, and (iii) Exit, the functions and the locations of which are similar to the corresponding instructions in the SCFC method. The CFCME delivers better an Evaluation Factor (EF) parameter in comparison with the other well-known software-based methods for multitask processing environments (CFCSS [17], ECCA [25], CEDA [27], and [28] method).

### 3.3. A Brief Review of the CPD Method

The CPD (Critical Path Duplication) [16] method is a data error detection method applicable in both single task and multitask processing environments. In well-known data error detection methods such as Full Duplication method, software redundancy is applied to the whole of the program and the comparison instruction for data error detection is placed at the end of the original and the duplicated sections. Any mismatch indicates an error. The difference between various data error detection methods is in where the comparison instructions are placed, the types of data diversity, and the redundancy section areas. In the CPD method, for decreasing the memory and the performance overheads and for keeping the Evaluation Factor high, the redundancy is applied only to the critical path of the Data Flow Graph (DFG) of the program. The DFG is extracted from the operand and the operator interactions. The critical path in the CPD method is a path, the running time of which is larger than the other paths of the DFG. Therefore, the duplication of software redundancy is applied to only this path. The comparison instruction compares the final outputs of the original critical path with the duplicated version of the critical path. Any mismatch generates an error signal. This signal shows a data error.

## 4. The Significance of Investigating Soft Error Management at Operating System Level

It should be noted that the operating system is a program itself. The difference between the operating system and the other programs lies in the fact that the operating system is a program that manages other programs and has critical tasks such as memory management and scheduling. However, the operating system itself needs a processor. The operating system is run on the same processor as the application program and manages the order of running of other programs and performs other management tasks. Therefore, the processor time used is mainly due to the application programs; the time spent by the operating system is generally very low. This could be the reason why researches have not, to date, focused on operating system level reliability. However, this assumption is not very logical. Although the probability of SEU occurrence on registers that belong to operating system is low, but the impacts of error occurring in these components are higher than the application level components. In the reliability analysis of a system, not only should the risk probability of an event be considered but also the effect of that event and the propagation of the resulting risks must be investigated.

## 5. Experimental Results

In this section, the test environment is explained and the experimental results are given.

For analyzing the effects of soft errors on operating system level, the following modules are utilized.

- A Personal Computer (PC): it is used for the development of the test programs or the compilation of the benchmark programs. The PC utilized for experiments has Intel (R) Core (TM) i5 CPU with 4 GB RAM and Ubuntu 11.04 operating system.
- A Background Debug Module (BDM): this component is a programming tool that can be used for debugging and fault injection. It is a tool which Motorola Corporation has placed it in its microprocessors and microcontrollers [11–16].
- An evaluation board (PhyCore-MPC555): this board is a product of a PHYTECH technology holding company.
- A Real Time Operating System (RTOS): for experiments, MicroC/OS-ii RTOS is utilized. This RTOS is a preemptive and multitask operating system.

For analyzing the impacts of soft errors on the components of the operating system kernel, some benchmark applications were run on the application level. Then, fault injections were done into the operating system kernel. In this way, the fault effects on both the operating system and the application level could be monitored. The utilized fault model in all experiments was SEU and the location of occurrence was the processor registers (address registers, data registers, and program counter and status register).

### 5.1. Evaluation of Applying the SCFC Method on Operating System Kernel

For analyzing the efficiency of SCFC on the modules of the operating system kernel, four application benchmarks were run at application level: (i) bubble Sort, (ii) quick sort, (iii) matrix multiplication, and (iv) linked list insertion. After this, 1200 faults were injected on Memory Manager kernel component. For the injection of faults on this component, the related data and the address registers to this component are manipulated.  Table 1 shows the fault injection results on Memory Manager without the use SCFC method and Table 2 shows the same results after applying the SCFC on this kernel component. The terminology used in the tables and the rest of the paper is as follows:

- CR (Correct Result): the fault does not change the final result of the program,
- OS (operating system): the fault is detected by operating system and its exceptions,
- WR (Wrong Result): the fault changes the final result of the program and produces a wrong output,
- TO (Time Out): the fault changes the program execution time and it does not end in a specified amount of time,
- SD (Software Detection): the fault is detected by the instructions that are used for Control Flow checking.

**Figure 4:**
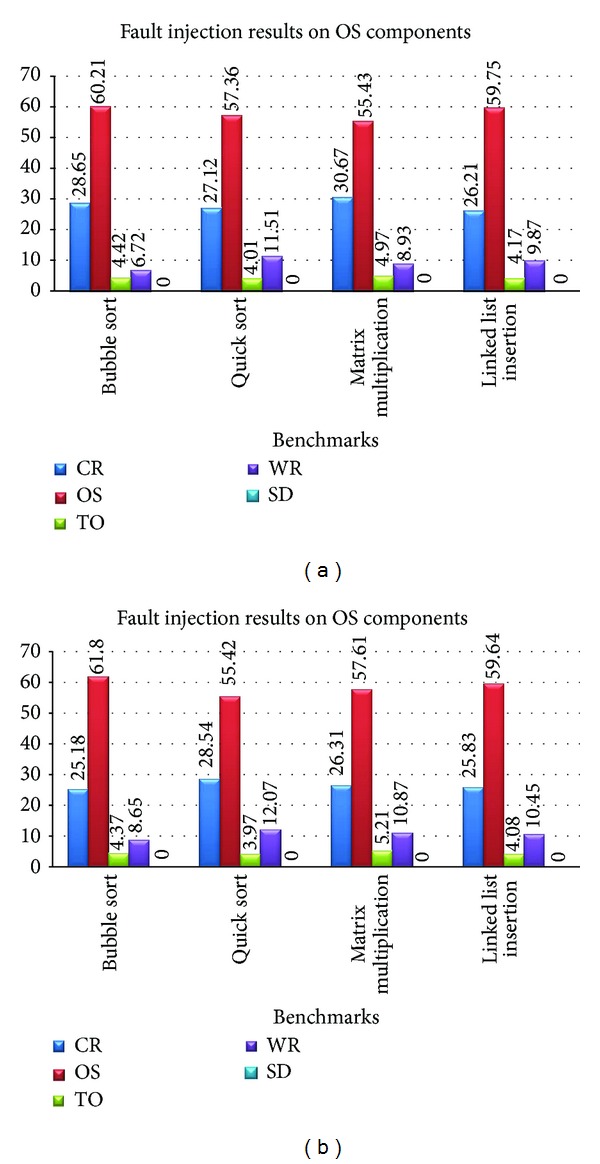
The average results from Tables 1 and 2.

It can be deduced from Tables 1 and 2 that the fault injection affects not only the kernel module but also the application benchmarks. In other words, a fault at the operating system level propagates to the other level (application). Table 2 shows that after applying the SCFC on the kernel components, the Wrong Results percentage is reduced and the Correct Result percentage is increased. Therefore, an increase in the fault tolerance of a kernel component increases the fault tolerance at the application indirectly.  Figure 4 shows the average results of Tables 1 and 2. As it can be seen from this figure, the percentage of Wrong Result before applying the SCFC method is %10.51 and this value is decreased to %9.25 after its application. The benchmark applications do not use the SCFC method. Therefore, the Software Detection percentage is in both cases zero.

This experiment shows that the injection of faults into the operating system can affect the whole components of the system. Although the probability of the occurrence of an error in the operating system is low, its effects are very dramatic.

### 5.2. Evaluation of Applying the CFCME on Operating System Kernel

For analyzing the efficiency CFCME on operating system kernel modules, similar to SCFC method, four application benchmarks were run in application level: (i) bubble sort, (ii) quick sort, (iii) matrix multiplication, and (iv) linked list insertion. As mentioned before, the CFCME is a CFC method for multitask processing environment. Therefore, test environment for this method has more than one component. In this experiment, three MicroC/OS-ii kernel components were selected: (i) Memory Manager, (ii) Process Manager, and (iii) Time Manager. Similar to the SCFC evaluation on the kernel component, in this process, application benchmarks were run firstly. Then 4800 SEU faults are injected on the kernel components.

**Figure 5:**
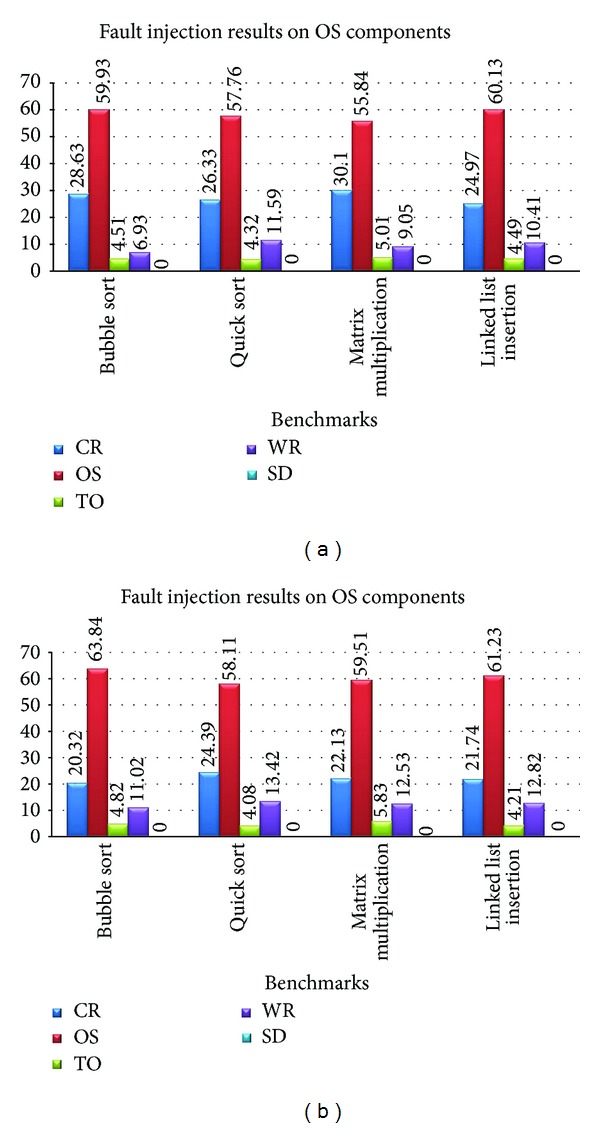
The average results from Tables 3 and 4.

Table 3 shows the fault injection results on the kernel components without applying the CFCME on them and Table 4 shows the same results after the application of the CFCME. The results of Tables 3 and 4 prove the claim of the previous section. As it can be seen from these two tables, after the application of the CFCME on the kernel components, the WR percentage is reduced and CR percentage is increased.  Figure 5 shows the average results of Tables 3 and 4. As it can be seen in this figure, Wrong Result percentage before applying the CFCME method is %12.45 and this value is decreased to %9.50 after applying the CFCME. It can be estimated that if more kernel components are selected, fault propagation to application level will increase. Therefore, applying CFCME method to the kernel components can avoid these errors both for the kernel and the application components.

### 5.3. Evaluation of Applying the CPD on Operating System Kernel

For analyzing the CPD efficiency on the operating system kernel modules, similar to SCFC and CFCME methods, four application benchmarks are run in application level. In this experiment, Core component (os_cpu_c.c file that has about 500 lines of code) of MicroC/OS-ii is selected for fault injection operation.  Table 5 shows the fault injection results on the kernel components without applying the CPD on Core component and Table 6 shows the same results after applying the CPD on this kernel component.

**Figure 6:**
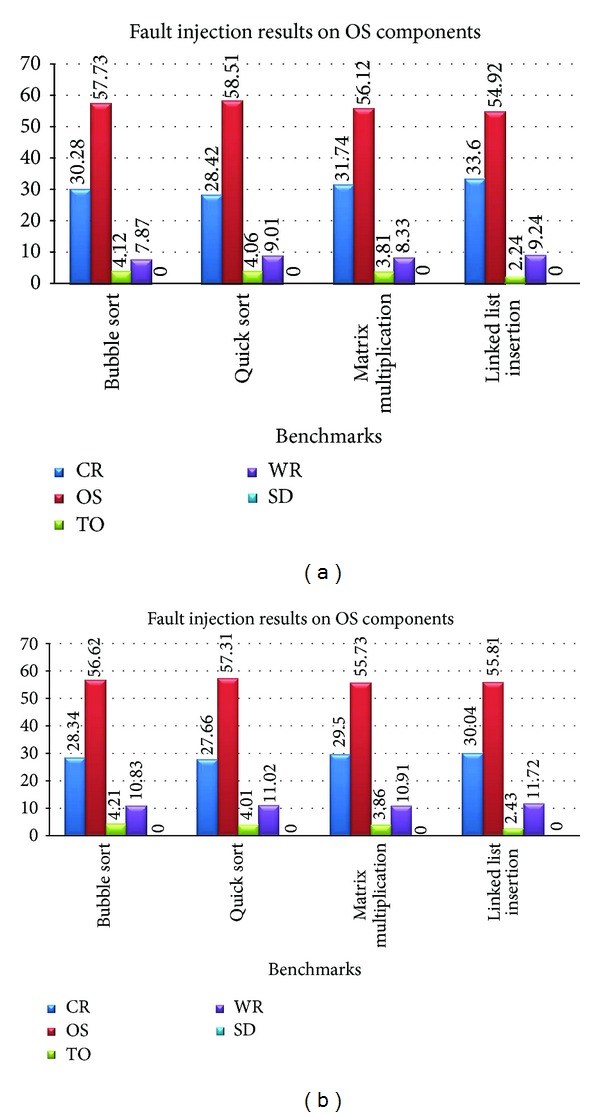
The average results from Tables 5 and 6.

Figure 6 shows the average results of Tables 5 and 6. As it can be seen in this figure, Wrong Result percentage before applying the CPD method is %11.2 and this value is decreased to %8.61 after applying the CPD. Therefore, the results of the previous section are proved here again.

## 6. Conclusions

The electronic equipment operating in harsh industrial environment is subjected to various threats. The most important threat to the proper behavior of the equipment is radiations. Electronic system designers have two options for selecting the equipment to handle the required operations in such a environment: High Reliability equipment and COTS equipment. Although the High Reliability equipment has high fault tolerance capability against transient faults, it has lower performance in comparison with COTS equipment. Moreover, the High Reliability equipment is a costly option and cost is an important factor in many applications that do not have a large budget. On the other side, the COST option may not meet the reliability requirements. Therefore, designers should improve the reliability of the equipment by the use of different hardware and/or software methods, the endeavor being to harden the equipment against CFE and data errors. To date, researchers have concentrated on managing these errors at application and hardware levels. In this paper, the effects of transient faults on operation system level are investigated. It is shown that the fault occurrence in operating system level has more dramatic impacts in comparison with the fault occurrence at other levels, due to the propagation of faults from this level to the other levels. The operating system components have strict relations with the components of the other levels. The experimental results of the paper have shown that by providing tolerance to the operating system kernel components against transient faults, both the operating system and the application components gain resilience and the percentage of Wrong Result in the application level components is indirectly reduced. It is to be noted that the figure for Wrong Result is inversely proportional to fault coverage. By decreasing the WR percentage, fault coverage percentage is increased. It should be stressed that even a small percentage change in fault coverage is significant.

## Figures and Tables

**Table 1:** Fault injection results on Memory Manager without SCFC.

Benchmark/Fault Injection	Without SCFC (%)				
	CR	OS	TO	WR	SD
Bubble Sort	25.18	61.80	4.37	8.65	0.00
Quick Sort	28.54	55.42	3.97	12.07	0.00
Matrix Multiplication	26.31	57.61	5.21	10.87	0.00
Linked List Insertion	25.83	59.64	4.08	10.45	0.00

**Table 2:** Fault injection results on Memory Manager with SCFC.

Benchmark/Fault Injection	Without SCFC (%)				
	CR	OS	TO	WR	SD
Bubble Sort	28.65	60.21	4.40	6.72	0.00
Quick Sort	27.12	57.36	4.01	11.51	0.00
Matrix Multiplication	30.67	55.43	4.97	8.93	0.00
Linked List Insertion	26.21	59.75	4.17	9.87	0.00

**Table 3:** Fault injection results on kernel components without CFCME.

Benchmark/fault injection	Without CFCME (%)				
	CR	OS	TO	WR	SD
Bubble sort	20.32	63.84	4.82	11.02	0.00
Quick sort	24.39	58.11	4.08	13.42	0.00
Matrix multiplication	22.13	59.51	5.83	12.53	0.00
Linked list insertion	21.74	61.23	4.21	12.82	0.00

**Table 4:** Fault injection results on kernel components with CFCME.

Benchmark/fault injection	Without CFCME (%)				
	CR	OS	TO	WR	SD
Bubble sort	28.63	59.93	4.51	6.93	0.00
Quick sort	26.33	57.76	4.32	11.59	0.00
Matrix multiplication	30.10	55.84	5.01	9.05	0.00
Linked list insertion	24.97	60.13	4.49	10.41	0.00

**Table 5:** Fault injection results on kernel components without CPD on core component.

Benchmark/fault injection	Without CPD (%)				
	CR	OS	TO	WR	SD
Bubble sort	28.34	56.62	4.21	10.83	0.00
Quick sort	27.66	57.31	4.01	11.02	0.00
Matrix multiplication	29.50	55.73	3.86	10.91	0.00
Linked list insertion	30.04	55.81	2.43	11.72	0.00

**Table 6:** Fault injection results on kernel components with CPD on Core component.

Benchmark/fault injection	With CPD (%)				
	CR	OS	TO	WR	SD
Bubble sort	30.28	57.73	4.12	7.87	0.00
Quick sort	28.42	58.51	4.06	9.01	0.00
Matrix multiplication	31.74	56.12	3.81	8.33	0.00
Linked list insertion	33.60	54.92	2.24	9.24	0.00
